# Young witnesses of intimate partner violence: screening and intervention

**DOI:** 10.1080/16549716.2019.1638054

**Published:** 2019-07-10

**Authors:** Lise-Lott Rydström, Maigun Edhborg, Lisa Ring Jakobsson, Zarina Nahar Kabir

**Affiliations:** Department of Neurobiology, Care Sciences and Society (NVS), Karolinska Institutet, Huddinge, Sweden

**Keywords:** IPV, children, witnessing violence

## Abstract

Intimate partner violence is a public health problem worldwide. Many children witness intimate partner violence at home and are affected by it. Regardless of the degree of exposure, children growing up in violent homes experience negative effects in the form of externalising behaviour and internalising symptoms which call for targeted interventions for children. The aim of the study is to map i) the available methods of detecting child and adolescent witnesses of intimate partner violence and ii) the interventions to support them. Three databases, PubMed, PsychInfo and Social Services Abstracts, were searched for scientific publications spanning over 20 years (1997–2017). This resulted in 2,406 publications of which 15 were finally selected after screening. Analysis of the articles resulted in three categories. The process of detecting children and adolescents who witnessed IPV in their homes varied in the included studies. The children were most commonly identified through their mother or other caregivers. Very few studies were based on children’s own reporting of their experiences, but were rather based on the mothers’ proxy reports. Studies distinguishing between the different forms of violence witnessed by children were few. It was uncommon that children were directly identified or screened for witnessing IPV in the family.

## Background

Intimate partner violence (IPV) defined as both acts and threats of physical, sexual, psychological and emotional violence perpetrated by a current or former intimate partner [1] is a public health problem worldwide [2]. As IPV is commonplace, many children are exposed to IPV as witnesses, since the violence takes place in the families’ homes. The prevalence of children’s exposure to IPV in the USA has been estimated to be 10–20% [3]. In Sweden 8–10% of the children in upper secondary school have witnessed at least one incidence of IPV during their upbringing [4]. IPV can be highly traumatic because children are close to the violence and thus experience severe posttraumatic stress disorder (PTSD) [5]. Regardless of degree of exposure to IPV, children growing up in violent homes experience more emotional, behavioural, social and cognitive problems and more symptoms, such as depression, anxiety, conduct disorder, aggression and attention-deficit-hyperactivity disorders (ADHD) than children growing up in nonviolent homes [6]. Children witnessing IPV react differently and express this in diverse ways with externalising behaviour and internalising symptoms [7]. It is more common that boys express themselves through externalising behaviour e.g. aggressive behaviour, rule-breaking behaviour, and substance abuse [7]. Girls, on the other hand, more often show internalising symptoms [8] e.g. anxiety/depression [7] as a symptom of being a witness of IPV. Experiencing multiple forms of violence e.g. witnessing IPV, being exposed to child abuse themselves, or being exposed to both family and community violence, were related to trauma more frequently than experiencing one single type of violence repeatedly [9].

As the women’s movement in the USA gathered momentum during the late 1960s and 1970s, the focus on women as victims of IPV and domestic violence became apparent. It was not until the 1990s that the impact of domestic violence on children received serious attention [9]. Research indicates that 40– 60% of the children witnessing IPV require treatment interventions [9]. This magnitude indicates the importance of effective, clinical and community-based interventions for children and adolescents [10]. Although some interventions designed for children exposed to IPV are available as clinical treatment, community based interventions are rare [11]. The aim of this paper is to map i) the available methods of detecting child and adolescent witnesses of intimate partner violence and ii) the interventions to support them.

## Method

**Figure 1.**
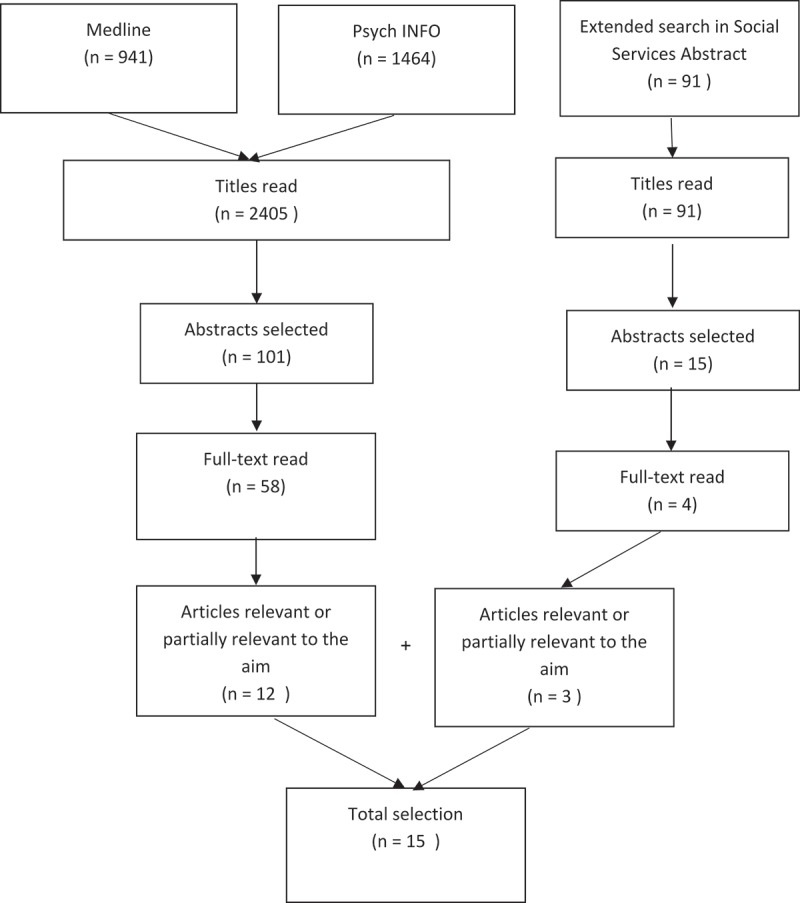
Process chart showing searches in databases.

The population of interest in this study was children aged 13–18 years who witnessed intimate partner violence (IPV) in the family. With the above objectives in mind, where IPV was the main exposure, a search of literature was conducted in September 2017, spanning over the previous 20 years in the databases Medline and Psych Info. The terms used in the search were: crime victims, exposure to violence, intimate partner violence, domestic violence, disclosure and adolescent, using truncations, AND, OR to combine the keywords. The search yielded 941 articles in Medline and 1464 in Psych INFO. First, all titles (2405) were read, of which 101 were found to be of interest in relation to the study objectives. Next, 101 abstracts were read and reduced to 58 articles of interest for the study. These 58 articles were read and only 12 of the articles were found to partially address the research objectives (Figure 1). Very few articles were found which specifically focused on detecting children and adolescents exposed to IPV in their homes and interventions for them. Therefore, a new search, using the same keywords, in the database Social Services Abstracts, which has a social focus, was conducted. The search resulted in 91 hits. Only three of the 91 articles were found to match the aim. What began as a systematic literature review was assessed to be best suited as a commentary by the authors due to the small number of articles that were found which addressed the study aims. Thus, finally15 articles were analysed. The articles were analysed using a modified version of content analysis [12]. The findings were broadly classified into three categories: 1) IPV rarely clearly classified as the main exposure, 2) Detecting child witnesses of IPV, and 3) Children not direct target group of interventions.

## Results

### IPV rarely clearly classified as the main exposure

One of the difficulties of conducting the analysis for a literature review was the diffuse definition of IPV as the main exposure. In most of the cases, IPV was not clearly classified, but rather combined with other violence, such as community, school and domestic violence including violence against siblings [13–16]. In addition, some articles did not distinguish between children experiencing family violence or witnessing [14,17–20]. Only in three studies [21–23], was IPV clearly assessed. In these studies, different types of IPV were specified, i.e. physical, psychological and sexual.

### Detecting child witnesses of IPV

The process of detecting children and adolescents (henceforth referred to as children) who witnessed IPV in their homes varied in the included studies. The children were most commonly identified through their mother or other caregivers (in 11 out of 15 articles) [13,14,17–25]. In six of the studies, the children were identified as witnesses only when their mothers reached out for help by contacting community services [13,14,17,19–21], for example, community based non-profit shelters, transitional housing [18], gender violence centres or emergency departments after being subjected to domestic abuse [25]. In some studies [14,18,21] child witnesses of IPV were identified through different types of questionnaire, such as the Conflict Tactics Scale, administered to the mothers. Further, child witnesses of IPV were found when their mothers were reported to the authorities for maltreatment of their children [21], if the children showed mental health problems, such as PTSD symptoms [14] or if the mothers contacted mental health services for themselves [15,17]. Another way of identifying the children was through screening in schools by self-reported questionnaires that children themselves filled in [16,26]. In one study, teachers identified students with aggressive behaviour and screened them for exposure to community and domestic violence [27]. In only two studies [16,26] were children approached directly to assess if they witnessed IPV in their families.

### Children not direct target group of interventions

Of the studies [13,14,17,18,21,24,25,27] that described interventions, none directly targeted the children themselves as the main focus of the interventions. Some interventions were created to support both the child witness of IPV and the mother/caregiver who was subjected to it [13,16]. In one study, different therapies were available for both the parents and the children, which also included group therapy for children witnessing domestic violence [25]. In one of these studies all information was gathered from the parents regarding children aged 3–18 years old [13]. Further, two studies focused on the parents or mother of the child in order improve parental practices [21,27].

## Conclusion

It is well established that the consequences of witnessing IPV might result in serious health problems amongst children and adolescents, including behavioural disorders and emotional disorders, as well as PTSD and social and academic problems. Additionally, perpetrators of IPV are more likely to have witnessed IPV as a child than non-perpetrators. Despite the knowledge that children suffer from these kinds of symptoms when witnessing IPV, interventions targeting children with the exposure is scarce. Most research is based on mother’s reporting of children’s exposure to IPV in the family.

In most of the articles that were reviewed, children were not directly identified or screened for witnessing IPV in the family, instead mothers were interviewed about their experience of IPV. Children were thus indirectly identified as being exposed to IPV in the family through reporting by those who were subjected to IPV.

In the reviewed studies, IPV in the family was often classified in the broad category of family violence which could include child abuse, sibling abuse, etc. Some studies included exposure to community violence in addition to family or domestic violence. Unless IPV is clearly classified as the exposure and child witnesses of IPV identified as the target group neither screening of these children nor targeted intervention can be effective.

Since children spend a substantial amount of their time in school, screening at the school those witnessing intimate partner violence might be considered. The school nurse can be a potential resource person using tools to capture this group of children and help them get in touch with professionals who may be able to support them through various interventions. As long as the voices of children and adolescents continue to be unheard it can be difficult to target effective interventions. Also, interventions need to be tailored in terms of the different types of violence that children are exposed to, i.e. community violence, domestic violence, violence in school, etc.

## Paper context

Of the 17 Sustainable Development Goals declared by the United Nations which are to be achieved by year 2030, a specific target (5.3) of Goal 5 is to eliminate violence against women and girls. Exposure to intimate partner violence (IPV) at home negatively impacts children’s mental health and their own attitude towards gender-based violence. Early interventions to support child witnesses of IPV at home can be critical in providing them and their communities with a healthy future. This article focused on mapping available methods for screening child witnesses of IPV and interventions to support them.
